# Engagement With Professional Stakeholders in Healthcare Research—The Case of the Dementia PersonAlised Care Team (D‐PACT) Project in the United Kingdom

**DOI:** 10.1111/hex.70133

**Published:** 2024-12-24

**Authors:** Basharat Hussain, Hannah Wheat, Tomasina M. Oh, Richard Byng

**Affiliations:** ^1^ Community and Primary Care Research Group University of Plymouth Plymouth UK

**Keywords:** dementia, engagement, evaluation, healthcare, involvement, professional stakeholders, professional stakeholders

## Abstract

**Introduction:**

In this viewpoint we highlight a gap in the literature relating to the involvement of professional stakeholders in healthcare evaluation research.

**Method:**

Using the Dementia—PersonAlised Care Team (D‐PACT) project as an example, we illustrate how professional stakeholder work can serve various functions, from understanding commissioning and policy context to contributing to detail of intervention components.

**Outcome:**

We argue that identifying these project‐specific functions can help researchers to effectively plan when, how and for whom they will engage in professional stakeholder work across the course of an evaluation. In addition, we call for further evidence‐based guidance and sufficient allocation of resources (provided by those funding research projects) to support effective stakeholder work.

**Conclusion:**

Such support will not only enhance evaluation findings but also promote continued learning on best practice for professional stakeholder work.

**Patient or Public Contribution:**

Public and patient involvement contributors were involved in the main D‐PACT study design, development of data collection tools and interpretation of study findings.

The importance of stakeholder engagement in health research is well recognised [1]. In the stakeholder engagement literature, there are two broad stakeholder categories, including patients, carers and lay public, and professionals working in various roles (e.g., researchers, policy makers, executives, managers, practitioners, support workers). The Medical Research Council (MRC) complex intervention framework [2] recommends engaging professional (as well as lay) stakeholders in the development, implementation and evaluation of healthcare interventions. Our focus in this commentary is on the involvement of professional stakeholders employed by health policy, planning and service delivery organisations in the public, volunteer, and private sector. There is a good volume of literature around the identification, engagement, evaluation and reporting of the patients/carers/lay public category. However, a rapid scoping review of existing literature relating to this topic, and correspondence with leaders in stakeholder work, suggest there is limited guidance professional stakeholder work for researchers wanting to meaningfully engage with professional stakeholders as well as report engagement work [3]. We define professional stakeholder work as activities related to the recruitment of professional stakeholders, and the collection and integration of stakeholders’ feedback in a research project.

The Dementia‐PersonAlised Care Team project (D‐PACT) is a 5‐year project, funded by the United Kingdom (UK) National Institute for Health and Care Research (NIHR) Programme Grant for Applied Research (PGfAR). The project aims to develop and evaluate a primary care‐based model of post‐diagnostic support for people with dementia and their carers. The support centres on what is important to the people receiving the support, and is delivered by dementia support workers, embedded within general practice surgeries. The aims of the (realist) D‐PACT evaluation [4], which is due to be completed in July 2024, are:

I. To better understand how the D‐PACT intervention is—and should be—delivered in varied settings (including to different communities) and use this knowledge to further refine the D‐PACT programme theory,
II. To better understand how best to support the practitioners delivering the intervention and use this knowledge to further refine the D‐PACT programme theory,
III. To explore the potential value and impact of the D‐PACT intervention,
IV. To contribute to the methodological development of community‐based dementia studies.

Our intention was to engage professional stakeholders at each stage of the evaluation to help fulfil the above‐mentioned aims. However, partly due to an absence of established guidelines, it was challenging for us to plan and manage this part of the evaluation. A key step that expedited progress was the identification of key functions that stakeholder work could serve throughout the evaluation [5]. Once functions were established, it was easier to develop stakeholder plans relating to who to involve; how to make and sustain contact with them; what activities to involve them in and when these activities would need to occur.

Our aim for this viewpoint is to promote further discussion on the importance of planning professional stakeholder work within evaluations. Using Dd‐PACT as a case study example, we briefly share what type of functions stakeholder work within an evaluation can serve. It is worth mentioning that while we focus on professional stakeholder activities here, most of these were also functions guiding stakeholder work with people with lived experience of dementia.

There were six key ‘functions’ for professional stakeholder work within the D‐PACT evaluation, each of these were informed by the evaluation aims:

I. *To co‐develop the intervention and practitioner support package*
  D‐PACT's intervention content was derived from multiple sources. We recognised that in addition to people with lived experience, dementia practitioners have valuable expertise that should be used to inform new models of care. Consequently, one stakeholder function was for practitioners to work with researchers to reflect and re‐design elements of the prototype intervention and practitioner support package. We hoped this collaboration would enhance the effectiveness, feasibility, and acceptability of these resources.
II. *Facilitating evaluation/research processes*
  To ensure our evaluation processes were as inclusive and effective as possible, we wanted to co‐design them with professional stakeholders. Predominantly (but not exclusively), this support related to our recruitment processes (making sure consent procedures were inclusive and our approaches to engaging with underserved communities were suitable). For stakeholder work to serve this function, we recognised a need to engage with: i), leaders within underserved communities, and with ii), researchers who had recruited people from these communities and had developed their own inclusive recruitment processes for dementia research.
III. *Intervention implementation facilitation*
  We were aware that we may come across unexpected problems when introducing a new intervention into the complex dynamic health and social care system. To help address such problems in a timely and effective way, we felt we would benefit from guidance from stakeholders with experience of working in such settings. As a result, we planned to maintain engagement with professionals working in various roles in various health care settings (e.g. primary network leaders; practice managers; roles associated with data governance and other dementia health care roles within various services/sectors).
IV. *Interpretation of value and impact*
  To ensure our evaluation findings were as valuable as possible, we decided it would be helpful to ask relevant others to provide their own interpretations of our data, to check or expand our own analyses. As a result, it became necessary for our stakeholder group to include a range of professional stakeholders with experience of delivering, managing and developing dementia healthcare strategies.
V. *Sustainability*
  As it is often difficult to sustain a health care intervention locally (6) (even one that has been shown to be successful) once a research project comes to an end, we determined that it would be important to work with professional stakeholders who could potentially provide a means for sustaining the model, or at least ensuring key findings from the evaluation were used in new dementia models/strategies. As a result, we sought to include members of our local Integrated Care Boards (ICBs) and a large‐scale dementia organisation (which fund their own dementia support workers) within our stakeholder group, to consider options for continuing D‐PACT support worker roles.
VI. *Dissemination*
  We recognised that professionals working within key dementia/health care organisations at a local and national level would be best placed to advise us on how best to disseminate our findings. We therefore worked to ensure there was sufficient representation from members of such groups within our stakeholder work, able to advise on outlets, format and content beyond research journals.

Figure 1 illustrates the functions, aim of stakeholder work in each function, and the groups involved.

**Figure 1:**
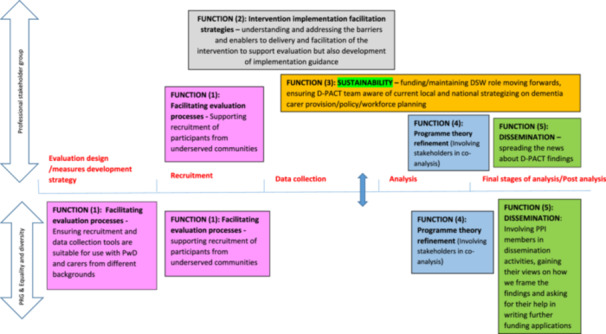
Stakeholders functions and their aims in D‐PACT.

The process of outreaching and recruiting professional stakeholders to join the research effort, including eligibility criteria and screening process is detailed below:

**1. Recruitment of professional stakeholders**

The eligibility criteria included any UK based professional with interest in dementia care working in the primary care setting (e.g., nurses, family doctors, paid carers, rehabilitation specialists, community support worker, social prescriber, paramedics), as well as ability to contribute to at least one of the six functions of D‐PACT.

We used range of strategies to recruit professional stakeholders. These included:

a. Recruiting through professional networks known to the D‐PACT team
b. First author screening for dementia researchers in the United Kingdom on Google. Following this, we contacted researchers with relevant research interest such as dementia care in the primary care setting.
c. Sending an email invite to ‘CHAIN’ (Contact, Help, Advice and Information Network) offering professionals in CHAIN network to join D‐PACT professional stakeholders group. CHAIN is a UK based online mutual support platform for health and social care professionals. Following this email, we received interest from a range of professionals including researchers, practitioners and volunteers.

**2. Training provided:** The D‐PACT team arranged several face‐to‐face and online meetings for the recruited professionals. Initially, in these meetings, the team introduced the D‐PACT project. Following this introduction, more focused meetings were held where each D‐PACT function was introduced in more details with guidelines on how professionals can contribute, for example, data analysis, dissemination.

**3. Barriers and challenges:** The main barrier in D‐PACT professional stakeholders work was the lack of any guidelines on how to carry out professional stakeholders work. We also encountered challenges in recruiting professionals, especially from local NHS providers as they were overstretched and had very limited time during and following the Covid‐19 pandemic. In addition, we struggled to engage professionals from Black and Minority Ethnicity (BME) volunteer sector organisations. Recruiting the required number of stakeholders for specific D‐PACT functions was particularly difficult, for example for the D‐PACT analysis function, we could only recruit one person. Furthermore, arranging meetings with diverse professional stakeholders on a mutually agreed time was a challenge.

We identified what resources may be needed, how resource use could be calculated, and what the impact of limited resources may be on engaging professional stakeholders. These are detailed below:

- Due to limited staff and financial resources, we were unable to undertake professional stakeholder work as originally planned. Professional stakeholder work required significant effort in terms of recruitment, their training and engagement. This mean dedicated staff resources are required to focus on this work. Professional stakeholder work also requires financial support so that professionals who engaged with the work are paid for their time; similar to paying PPIE members. Professionals in health and social care are already stretched in their work and asking them to contribute to a research project like D‐PACT without compensation for their time does not appear to be an attractive or fair option.
- Staff resources could be calculated on the basis of planned professional stakeholder activities. These activities, for example, include how broad the recruitment of professional stakeholders would be, how many meetings are planned. Financial resources could be calculated on the basis of per hour rate of professional from different category. For example, per hour payment rate could be calculated based on monthly salary of professionals, such as a GP.
- Impact of limited resources meant that professional stakeholders were not effectively involved in the intervention development and testing (trial) phase. This means that even if the intervention is effective, it might not be successful in the ‘real world’ as it has not been informed by those who would implement the intervention in practice, and have the experience and understanding of the context in which the intervention would be delivered.

## Conclusion

Within the evaluation we have documented what professional stakeholder work has been undertaken and what it led to. Ideally, we would have wanted to further conceptualise and evaluate such work but were unable to due to limited project resources. We recognise that stakeholder work has ambiguous boundaries, with for example ‘knowledge exchange’ where at the start we are ‘learning from’ and later in the programme ‘giving back’. In the future, we hope to see more transparency on how professional stakeholder work was undertaken and published guidelines informed by such insights. We would also hope to see increased awareness of how important (but time intensive) this work is within funding application guidelines. We would suggest that consideration of stakeholder functions is a helpful initial step toward planning any type of professional stakeholder work.

## Author Contributions

**Basharat Hussain:** writing–original draft, writing–review and editing. **Hannah Wheat:** conceptualisation, methodology, writing–review and editing, supervision. **Tomasina M. Oh:** writing–review and editing, supervision. **Richard Byng:** conceptualisation, methodology, supervision, funding acquisition, writing–review and editing; project administration.

## Ethics Statement

The authors have nothing to report.

## Consent

Has been declared by all authors.

## Conflicts of Interest

The authors declare no conflicts of interests.

## Data Availability

Data sharing is not applicable to this article as no data sets were generated or analysed during the current study.
